# Prevention and Control of Antimicrobial Resistant Healthcare-Associated Infections: The Microbiology Laboratory Rocks!

**DOI:** 10.3389/fmicb.2016.00855

**Published:** 2016-06-07

**Authors:** Alexandra S. Simões, Isabel Couto, Cristina Toscano, Elsa Gonçalves, Pedro Póvoa, Miguel Viveiros, Luís V. Lapão

**Affiliations:** ^1^Global Health and Tropical Medicine, Instituto de Higiene e Medicina Tropical, Universidade Nova de Lisboa, LisbonPortugal; ^2^Laboratório de Microbiologia Clínica e Biologia Molecular, Serviço de Patologia Clínica, Hospital de Egas Moniz, Centro Hospitalar de Lisboa Ocidental, LisbonPortugal; ^3^Centro de Estudos de Doenças Crónicas, NOVA Medical School/Faculdade de Ciências Médicas, Universidade Nova de Lisboa, LisbonPortugal; ^4^Unidade de Cuidados Intensivos Polivalente, Hospital de São Francisco Xavier, Centro Hospitalar de Lisboa Ocidental, LisbonPortugal; ^5^WHO Collaborating Center for Health Workforce Policy and Planning, Instituto de Higiene e Medicina Tropical, Universidade Nova de Lisboa, LisbonPortugal

**Keywords:** microbiology, healthcare-associated infections, antibiotics, antibiotic stewardship, information systems, prevention, control, communication

## Abstract

In Europe, each year, more than four milion patients acquire a healthcare-associated infection (HAI) and almost 40 thousand die as a direct consequence of it. Regardless of many stategies to prevent and control HAIs, they remain an important cause of morbidity and mortality worldwide with a significant economic impact: a recent estimate places it at the ten billion dollars/year. The control of HAIs requires a prompt and efficient identification of the etiological agent and a rapid communication with the clinician. The Microbiology Laboratory has a significant role in the prevention and control of these infections and is a key element of any Infection Control Program. The work of the Microbiology Laboratory covers microbial isolation and identification, determination of antimicrobial susceptibility patterns, epidemiological surveillance and outbreak detection, education, and report of quality assured results. In this paper we address the role and importance of the Microbiology Laboratory in the prevention and control of HAI and in Antibiotic Stewardship Programs and how it can be leveraged when combined with the use of information systems. Additionally, we critically review some challenges that the Microbiology Laboratory has to deal with, including the selection of analytic methods and the proper use of communication channels with other healthcare services.

## Introdution

Healthcare-associated infections (HAIs) are a significant cause of morbidity and mortality leading to 37,000 deaths/year in Europe^1^ and 75,000 deaths in USA in 2011 (Magill et al., 2014). The HAIs economic impact is also significant: about 9.8 billion dollars/year/USA for the five major infections (Zimlichman et al., 2013). Antibiotic resistance is one of the major problems associated with HAIs (Cosgrove, 2006; Neidell et al., 2012): the Centers for Disease Prevention and Control (CDC) estimates that over two million people/year acquire antibiotic resistant infections, and 23,000 die as a result of it (CDC, 2013). In Europe, 25,000 people/year die with drug-resistant infections (ECDC, 2009).

Prevention through education is the most used strategy for HAIs control: the benefits from prevention can be as high as 5.5 billion dollars (Zimlichman et al., 2013). Reinforcing hand washing, staff education, environmental cleaning practices, Antibiotic Stewardship Programs (ASPs) and improved communication systems are measures implemented worldwide to control HAIs. However, to efficiently control HAIs, clinicians need to act quickly, which implies gathering all relevant information about the infection as soon as possible. That is why the Microbiology Laboratory is so important in HAIs prevention and control since it is in the front line for the early identification of infection, characterization of antibiotic resistance patterns and recognition of outbreaks (Diekema and Saubolle, 2011; Davey et al., 2013).

Traditionally, the tasks of the Microbiology Laboratory are to isolate, identify and determine antibiotic susceptibility patterns of pathogens (Wilson and Spencer, 1999). However, its scope covers other areas that are critical for Infection Control Programs as well as to ASPs, which should ideally include a member of the Microbiology Laboratory staff (Kolmos, 1999; Benbachir, 2008; CDC, 2014). The success of these kind of programs depends largely on the active involvement of the Microbiology Laboratory in activities beyond the regular microbiology exams, namely in results report, surveillance, communication, and other daily routine tasks of Infection Control Teams (Kalenić and Budimir, 2009).

In this paper, we describe the multiple chores of the Microbiology Laboratory highlighting its importance in HAIs prevention and control, and in ASPs, especially when combined with properly designed information systems. We also review some of the problems that the Microbiology Laboratory has to deal with when assisting Infection Control Teams, including the selection of the most appropriate analytic methods to provide fast and accurate results.

## Tasks of the Microbiology Laboratory

### Microbial Isolation and Identification

The Microbiology Laboratory main task is to isolate and identify the infection etiological agent (Benbachir, 2008), using the most appropriate, rapid and accurate diagnostic method. To ensure this, the Microbiology Laboratory needs to keep up-to-date materials, culture media, reagents, equipment, identification methods and trained personnel (CDC, 2003). The staff needs continuous on-the-job training in microbiological techniques and to be updated on the internationally endorsed methods for isolation and characterization of pathogens (Pfaller and Herwaldt, 1997; Benbachir, 2008). In addition, external and internal quality control and assurance programs must be implemented to guarantee the quality of the results (Benbachir, 2008).

### Determination of Antimicrobial Susceptibility Patterns

The Microbiology Laboratory should provide frequently updated information on antimicrobial resistance patterns, essential to design appropriate hospital prescription guidelines, help clinicians to choose the most appropriate empiric therapy and to create a culture of patient safety (Pfaller and Herwaldt, 1997; Benbachir, 2008). This data can be analyzed in different perspectives, including infectious agent, specimen, ward, clinical specialty, antibiotics prescribed, or anatomic site of infection, among others (Pfaller and Herwaldt, 1997; Benbachir, 2008).

The availability of periodic reports on local antimicrobial resistance patterns is also relevant in ASPs, since it can be used to evaluate trends of antimicrobial resistance rates, to educate clinicians on optimal antimicrobial use and to assess the impact of prevention measures (Diekema and Saubolle, 2011).

### Report of the Results

The laboratory work is completed only when it is effectively reported (Newton and Novak-Weekley, 2011). All laboratory results (preliminary and final) should be reported as soon as possible to clinicians and Infection Control Teams (Kalenić and Budimir, 2009). Daily reports on significant microbiology results and periodic reports with frequency of isolated pathogens and prevalence of resistant microorganisms provide clinicians and Infection Control Teams with accurate and timely information, essential to follow trends of hospital infections and control urgent situations (Pfaller and Herwaldt, 1997; Benbachir, 2008).

These reports can be delivered through meetings, phone, information systems alerts, paper, or e-mail. However, making results accessible through an information system is an advantage by ensuring that all results are available in an organized, easily accessible, and timely manner, and also permits links to other surveillance data systems (Cantón, 2005). Information systems that incorporate information about the patient, disease, infectious agent and antimicrobial susceptibility are fundamental because they promote timely exchange of information between healthcare workers (Schreckenberger and Binnicker, 2011). For instance, ARTEMIS (Teodoro et al., 2012) and HAITool (Pinto et al., 2016) are good examples of this kind of systems. They analyze heterogeneous data sources and can be used to build antimicrobial resistance surveillance networks participating in the management and prevention of antibiotic resistant HAIs, and by doing so, optimizing human and economic resources^2^ (Teodoro et al., 2012). Some of these systems alert the clinician (or the pharmacist) when laboratory results reveal that the antibiotic(s) in use may not be optimal or when de-escalation treatment is indicated (Schreckenberger and Binnicker, 2011; Pinto et al., 2016). The “processed information” generated by these systems are very useful for surveillance purposes and in supporting and leveraging ASPs (Davey et al., 2013).

### Surveillance and Outbreak Detection

Surveillance enables the identification of infected patients, the origin of HAIs and to understand their paths of spread. Since most of this data comes from microbiological isolates and other laboratory identification tests (Emori and Gaynes, 1993; Peterson and Brossette, 2002), the Microbiology Laboratory has a central role not only on the surveillance and early detection of outbreaks but also on monitoring and reporting unusual laboratory results (e.g., clusters of pathogens, emergence of multidrug-resistant organisms, isolation of unusual pathogens).

To detect outbreaks early enough to mitigate their impact on morbidity and mortality is one of the major challenges of an efficient surveillance program (Diekema and Saubolle, 2011). The Microbiology Laboratory, in association with the Infections Control Team, is the first to detect an outbreak because unusual clusters of pathogens or resistance patterns are easily noticed (Arias, 2010). During an outbreak, these two entities have to work side by side to: (i) provide information on the epidemiology of the etiologic agent; (ii) identify and store the isolates involved for further testing; (iii) define/select appropriate selective isolation media and drug susceptibility testing (if needed/when applicable); (iv) perform the appropriate tests for strain typing (or provide its dispatch to a reference laboratory); (v) and perform supplemental microbiological surveillance of patients, personnel, or environmental sources of infection (Emori and Gaynes, 1993; Pfaller and Herwaldt, 1997; Arias, 2010).

As described above, the use of surveillance systems can enhance surveillance programs by aggregating all the information related with patient, disease, infectious agent and antimicrobial susceptibility, making easier outbreaks detection.

### Education

To maximize the efficacy of Infection Control programs, the Microbiology Laboratory should provide training and information on basic microbiology and biosafety for healthcare workers in areas such as: specimen collection, handling and transport, epidemiologically important pathogens vs. normal flora, colonization vs. infection, interpretation of microbiological results (Kalenić and Budimir, 2009). Written guidelines about sampling, handling and transport should be available in every ward, which can also include details on the tests available for proper isolation, identification, and typing of microorganisms (Grosek, 1999).

## Antibiotic Stewardship Programs

Antibiotic Stewardship Programss should be included in all HAIs prevention and control programs and are essential for gathering information about epidemiological and molecular markers of resistance, and changes in resistance patterns. They not only contribute to the optimization of antimicrobial therapy, by ensuring proper use (indication, dose, route of administration, and duration) and minimizing side effects, but also promote education on it (Davey et al., 2013). The adoption of these programs leads to a reduction in the prevalence of antimicrobial resistance and costs (Malani et al., 2012). These programs have been implemented throughout the world and there are guidelines and recommendations for their use in the USA (CDC, 2014; Fridkin et al., 2014) and Europe (Pina, 2002; Department of Health Advisory Committee on Antimicrobial Resistance and Healthcare Associated Infection, 2011; Lower et al., 2013; Simões et al., 2015).

Also here, computerized surveillance and decision support systems are a good support for ASPs, since have proven to be effective in the prescription errors reduction, medical care improvement and compliance with recommendations (Evans et al., 1998; Pestotnik, 2005).

**FIGURE 1:**
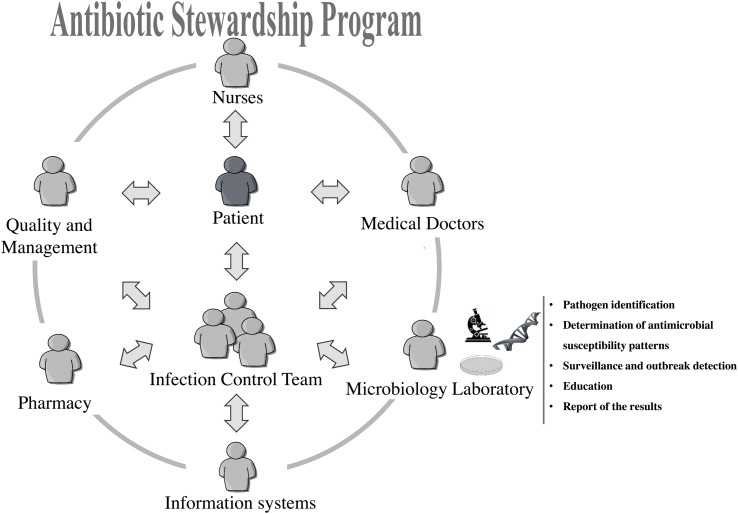
**Network of interactions in an Antibiotic Stewardship Program**.

A schematic representation of the network of interactions in ASPs is shown in **Figure 1**. The laboratory imparts an important function in ASPs, generating most of the relevant information needed to characterize the biology of the pathogen (and the hosts), namely its identification, antimicrobial susceptibility patterns and epidemiological connections. Therefore, it is recommended that all ASPs include a microbiologist (CDC, 2014). A good example of the role of Microbiology Laboratory in ASPs is described by MacKenzie et al. (2007) who demonstrated that hospitals with routine reports on antimicrobial susceptibility patterns for restricted antibiotics had lower usage (and misusage) of these antibiotics.

## Issues on the Laboratory Efficacy

### Sample’s Quality and Access to Clinical Data

The quality of the laboratory diagnosis is closely related with the quality of the collected samples (Benbachir, 2008). Samples should be taken on the correct time and from appropriate sites, using proper techniques and in amounts that makes possible to perform all the tests necessary for the isolation and identification (and other testing) of the pathogen (Department of Health Advisory Committee on Antimicrobial Resistance and Healthcare Associated Infection, 2011). Samples that are not properly collected and transported may lead to false results (Benbachir, 2008).

Relevant epidemiological and clinical information data are also important for accurate laboratory diagnosis. Every request for exam should include: name of the patient, name of the clinician in charge, location of the patient, date and time of specimen collection, short anamnesis including suspected diagnosis and underlying patient conditions and comorbidities (Benbachir, 2008; Kalenić and Budimir, 2009). The access to this pre-analytic data facilitates significantly the guidance toward pathogen detection and identification. These kind of information should be easily accessed throughout an information system.

### Isolation in Culture or Molecular Identification Methods, Which One to Choose?

The use of laboratory culture methods for isolation of pathogens followed by identification procedures (biochemical, molecular, serologic, or other) has been the gold standard in Medical Microbiology and continues to play a vital role in the overall management of infectious diseases. As an example, blood culture is still mandatory for isolation and identification of blood pathogens and guiding therapy (Book et al., 2013). However, isolation in culture has limitations, namely being time consuming (Singh et al., 2006). Results provided 48 to 72 h after the onset of the infection often have limited impact on therapy (Tenover, 2010) and the ability of the laboratory to provide results in a timely manner is essential.

Conversely, the major benefit of direct molecular methods is related to time saving. Using Polymerase Chain Reaction (PCR) based methods, results can be obtained within 6 to 8 h, reducing greatly the time-to-result and implementation of appropriate therapy (Book et al., 2013). Molecular methods have been described as a powerful tool against the spread of microorganisms in hospital environment, in particular during outbreaks (Singh et al., 2006), and many have been used to rapidly identify microorganisms (e.g., multiplex PCR, Real-time PCR, MALDI-TOF MS), to find resistance patterns (e.g., PCR/hybridization screening of resistance determinants) and to estimate epidemiological links between bacteria (e.g., pulsed-field gel electrophoresis, multi-locus sequence typing), enabling rapid and appropriate therapeutic responses (Cantón, 2005; Simões et al., 2011; Sabat et al., 2013; Patel, 2015; Srinivasan et al., 2015). More recently whole-genome sequencing (WGS) promises to transform Infection Control. WGS provides almost all the genetic information needed for epidemiological studies (Köser et al., 2012) and has been widely used to identify and control outbreaks of antibiotic resistant HAIs (Reuter et al., 2013; Price et al., 2013). WGS can be used to quickly identify pathogenic agents from specimens and unravel many genomic single nucleotide polymorphisms and markers for drug resistance, allowing the implementation of immediate and appropriate control measures (Coll et al., 2014). However, the translation of the large amount of data generated by WGS to the clinical utility is still under development (Burke and Korngiebel, 2015).

Molecular methods have been associated with global cost reduction, due to their high specificity, sensitivity and rapid turnaround (Cantón, 2005; Tenover, 2010; Currie, 2011). Combining molecular typing with surveillance programs was shown to be cost effective and result in significant reduction of HAIs rates (Hacek et al., 1999). More recently, the integration of rapid identifications methods with stewardship interventions has been described as a way to improve time to optimal antibiotic therapy, decrease length of hospital stay and reduce mortality and healthcare costs (Huang et al., 2013; Bauer et al., 2014; Perez et al., 2014).

However, molecular methods do not solve all the problems. Several authors have described drawbacks associated with molecular methods: limited number of detectable pathogens, possibility of false positives and complex sample preparation procedures (Laxminarayan et al., 2013). In addition, molecular methods are more expensive, require specialized equipment, and training (Morshed et al., 2007) and their value in diagnosis of some infections has not been fully proven. For instance, in a recent study testing the accuracy of Septi*F*ast multi-pathogens real-time PCR, authors concluded that, despite providing faster results, this method has limited utility in bloodstream infections, when compared with conventional blood culture (Warhurst et al., 2015). Additionally, there are several microorganisms for which isolation by culture methods is the most effective and recommend procedure especially when dealing with drug-resistant inducible geno/phenotypes.

The dispute on isolation in culture followed by identification versus direct identification from specimen by molecular methods seems endless and unnecessary since both methods can be used in concert. Several authors suggested a combination of culture and molecular methods in order to increase the rate, efficacy and accuracy of pathogen detection. (Dolinger and Jacobs, 2011; Brown-Elliott and Wallace, 2012; Huttunen et al., 2013). **Table 1** summarizes some pros and cons of both strategies.

**Table 1:** Comparison of culture and molecular identification methods.

	Culture methods	Molecular methods
Specificity	Moderate	High
Sensitivity	Low	High
Antimicrobial susceptibility	Isolates can be tested for susceptibility to relevant antibiotics	Allows the detection of some resistance markers without isolation/culturing
Amount needed to detected pathogens	High	Low
Time to obtain results	Long (especially for slow-growers organisms)	Short
Cost	Low	High (variable)
Detection of non-viable bacteria (patient in antibiotic treatment)	No	Yes
Equipment	Requires non-specialized equipment	Requires specialized equipment
Biosafety	Potential biosafety concerns	Minimizes biosafety concerns
Feasibility	Requires basic training No specialized workflow required	Requires advanced training Assays may not be commercially available
Others	Allows visual inspection of colony morphology Allows biochemical characterization of phenotype Limited potential for false positives and/or false negatives	Allows high resolution analysis Potential false positives (by cross-reaction with closely related species or contaminated amplicons) Potential false negatives (by inhibition components or target mutations)

*Adapted from (Dolinger and Jacobs, 2011).*

### Antimicrobial Resistance Surveillance

With the emergence and spread of antimicrobial resistant pathogens, antimicrobial resistance surveillance is becoming an important task of the Microbiology Laboratory. Antimicrobial resistance surveillance is an ongoing (and organized) data collection that after being analyzed and reported provides useful information for empirical antimicrobial therapy (Cornaglia et al., 2004).

Nevertheless, a good surveillance program is time consuming and involve dedicated human resources. In addition there are several challenges on data collection, management, analysis, interpretation and reporting. For instance, promoting the use of new and low-cost technologies to improve laboratory work and to prioritize which bacteria are most important to track are issues that should be addressed (Solomon and Ijaz, 2015). Regarding interpretation, uniformization is needed: currently, different guidelines and breakpoints for evaluation of antimicrobials susceptibility patterns values are adopted in the United States of America and within several European countries (Vernet et al., 2014). Finally, the results should be presented in formats easily understandable by the clinicians (Grundmann et al., 2011).

Information systems are a good way to keep-it working smoothly (Lapão, 2007). As stated above, surveillance information systems ensure that antimicrobial resistance related data are available and organized, making easy to report it retrospectively. In addition, surveillance information systems allow better antibiotic resistance management and help to provide evidence based results that can be used for the development of control policies (Evans et al., 1998; Pestotnik, 2005).

### Communication

Effective communication is critical for the Microbiology Laboratory procedures. Nevertheless, it can also be one of its major issues. Effective communication is the base of a healthy collaboration between laboratory, healthcare workers and Infection Control Teams (Kalenić and Budimir, 2009). The dialog between healthcare workers and laboratory staff must be easy and effective (Peterson et al., 2001). The existence of a dedicated laboratory staff element (privileged interlocutor) and the participation of microbiologists in regular clinician’s meetings is recommended. Efficient communication between clinicians and microbiologists about the presumptive diagnosis, accelerates the diagnosis and avoids problems with inappropriate specimens (Baron et al., 2013).

In order to facilitate communication, it is recommended that the director of the Microbiology Laboratory be a clinician or a laboratory scientist with expertise in infectious diseases and microbiology (Thomson et al., 2010) since their background on disease pathologic process facilitates the discussion of clinical cases. Additionally, information systems have an important role in communication within the hospitals by facilitating the exchange of clinical and microbiological relevant data between clinicians and laboratories (Lapão, 2007). However, it is important that information systems are defined and designed together with the healthcare workers in order to really improve communication, data quality, be useful on decision-making and be easy to use (Pinto et al., 2016).

Another reality that can affect the communication is the location of the Microbiology Laboratory. There are an increasing number of off-site laboratories providing services to hospitals and in the cases of in-house laboratories, they usually are in the basement or in an annex outside the hospital main building. It has been described that off-site laboratories delay and decrease communication, could jeopardizes infection diagnosis and treatment and weaken infection prevention and antibiotic stewardship infrastructures (Peterson et al., 2001; Dancer et al., 2015).

## Conclusion

The Microbiology Laboratory plays a key role in HAIs prevention and control. From ensuring appropriate specimen collection and transport, to the wise selection of isolation and identification methods and finally on the antibiotic therapy guidance plus effective report and communication of the results, the laboratory covers all important aspects of infection control process. The microbiologist is a fundamental and enriching member of ASPs and Infection Control Teams. The new identifications methods (including WGS) combined with the emergence of innovative and centralized information systems that integrate the microbiology results with clinical data will revolutionize HAIs prevention and control strategies, help decision-making and resolve some of the difficulties felt by the microbiologists.

## Author Contributions

ASS, IC, CT, EG, PP, MV, and LVL contributed to the draft and revision of the paper and approved the final version to be published.

## Conflict of Interest Statement

The authors declare that the research was conducted in the absence of any commercial or financial relationships that could be construed as a potential conflict of interest.

## Footnotes

1 http://ecdc.europa.eu/en/healthtopics/Healthcare-associated_infections

2 http://haitool.ihmt.unl.pt/
